# The performance of the SD BIOLINE Dengue DUO® rapid immunochromatographic test kit for the detection of NS1 antigen, IgM and IgG antibodies during a dengue type 1 epidemic in Jamaica

**DOI:** 10.1186/s12929-015-0164-9

**Published:** 2015-07-16

**Authors:** Ivan E. Vickers, Kevin M. Harvey, Michelle G. Brown, Kereann Nelson, Marion Bullock DuCasse, John F. Lindo

**Affiliations:** Department of Microbiology, The University of the West Indies, Kingston 7, Mona, Jamaica; Ministry of Health, Kingston, Jamaica

**Keywords:** Dengue, IgM, IgG, NS1, Antibody, Antigen, Sensitivity, Specificity, Positive predictive value, Negative predictive value

## Abstract

**Background:**

Dengue is an important mosquito-borne viral infection that affects millions of persons worldwide. Early diagnosis is necessary to effect appropriate management and decrease mortality. Immunochromatographic tests are advantageous in producing dengue test results within 30 min but these results should be sensitive and specific. In this study we evaluated the diagnostic performance of the SD BIOLINE Dengue DUO® rapid immunochromatographic test kit. A panel of 309 dengue and 30 non-dengue single serum samples characterized by using reference enzyme-linked immunosorbent assays (ELISAs) was used. These samples were received in the virology laboratory for routine testing during a dengue type 1 outbreak between October to December, 2012.

**Results:**

The overall diagnostic sensitivities of the SD BIOLINE Dengue DUO® rapid testfor IgM, IgG and NSI were 49.3 % (95 % CI: 41.3-57.4), 39.1 % (95 % CI: 33.3-45.2) and 90 % (95 % CI: 82.1-94.7), respectively. The IgM and IgG detection rates were significantly lower than that of the NSI (*p* < 0.001). However the combination of the IgM detection with NS1 detection or both NS1 and IgG resulted in a significant (*p* < 0.001) increase in sensitivity to 97.5 % (95 % CI: 92.9-99.2) and 98.9 % (95 % CI: 96.0-99.7), respectively. These higher sensitivities were achieved without any decrease in specificities.

**Conclusions:**

This study revealed that combining two or more parameters of the SD BIOLINE Dengue DUO® rapid kit significantly improved the sensitivity of diagnosis of dengue virus infection and supports its usefulness in the Jamaican setting.

## Background

Dengue is a viral infection caused by the dengue virus (DENV). DENV is a member of the *Flaviviridae* family of ribonucleic acid (RNA) viruses which also includes West Nile virus (WNV), Yellow fever virus (YFV) and Japanese encephalitis (JE) virus [1, 2]. There are four serotypes of dengue virus (DENV-1, DENV-2, DENV-3 and DENV-4) although recently a possible fifth serotype(DENV-5) was reported [3]. The virus is arthropod borne and is transmitted to humans by the bite of an infected female mosquito. The primary vector is the *Aedes aegypti* mosquito but other species such as *Aedes albopictus* and less commonly *Aedes polynesiensis* can also transmit the virus [4, 5]. Dengue occurs in tropical and subtropical regions of the world with endemicity in over 100 countries including Jamaica [2, 6–8]. Although dengue is endemic in the Americas, outbreaks generally recur with a 3 to 5 year cycle [8]. The last epidemic in Jamaica was in the year of 2012 and was caused by DENV-1 [9, 10].

The clinical manifestations of dengue usually follow an incubation period of 2–7 days and may include a wide variety of signs and symptoms [11]. According to the most recent classification by the World Health Organization (WHO) persons are classified as having dengue with or without warning signs or severe dengue [12]. The criteria for dengue without warning signs include fever and two of nausea and vomiting, rash, aches and pains, leucopenia and a positive tourniquet test. Warning signs include abdominal pain or tenderness, persistent vomiting, mucosal bleeding, among others. There is no vaccine or specific treatment for dengue but early diagnosis and supportive management can decrease the mortality of severe dengue disease [13].

The laboratory diagnosis of dengue includes virus isolation, serological and molecular techniques [5, 12, 14]. Viral isolation is generally time-consuming while molecular methods are expensive. Enzyme-linked immunosorbent assay (ELISA) is most often used in the diagnosis of dengue in Jamaica and other countries. These tests detect dengue specific antibodies such as immunoglobulin (Ig)-M, IgG, IgA or dengue antigens particularly non-structural (NS)-1 glycoproteins [15, 16]. More recently, rapid immunochromatographic tests (ICTs) have become available. The diagnostic performances of the dengue ICT kits have been noted to vary with different countries. We, therefore, sought to determine the performance characteristics of a rapid dengue ICT kit in Jamaica.

## Methods

### Study site

The study was conducted at the virology laboratory in the Department of Microbiology of the University Hospital of the West Indies (UHWI), a tertiary referral hospital, after ethical approval was obtained (ECP 181, 12/13). The virology laboratory is the reference laboratory for testing dengue virus in Jamaica and receives specimens from all 14 parishes of the island.

### Study design

A retrospective cross sectional design was used to screen archived single serum samples received in the virology laboratory with a request for dengue IgM antibody testing between October and December 2012. All samples were stored at −70 °C after routine diagnostic testing until included in this study for evaluation. The inclusion criteria for the sample selection were: presence of the date of onset of symptoms, presence of the date of collection of specimen and sufficient sample volume. A total of 339 of the 3402 archived single serum samples met the inclusion criteria and were selected. Demographic and clinical information were extracted from the hospital records.

### Dengue diagnostic tests

The dengue NS1 antigen ELISA (Standard Diagnostics Inc., Seoul, Korea) and the dengue IgM and IgG antibody capture ELISAs (Focus Diagnostics, Cypress, PA, USA) were used as the reference methods [16–18]. All reference testing procedures were performed and interpreted according to the manufacturers’ instructions except for the interpretation of the IgM assay. The IgM ELISA was interpreted as: − positive: index value ≥1.2; negative: index value <1.0; equivocal: index value >1.0 and <1.2. Samples (n = 28) that were repeatedly equivocal were excluded from analysis. The manufacturer’s instructions for the SD BIOLINE Dengue DUO® (SDB DD) NS1 Ag and IgG/IgM ICT were followed and are described previously [19]. Briefly, 100 μl and 10 μl of serum specimen were added to the sample well “S” of the NS1 Ag and IgM/IgG strips of the combo device, respectively. Four drops of assay diluents were added to the assay diluent well of the latter. Both strips of the device were read at 15–20 min.

### Classification of samples

The samples were classified as dengue, non-dengue, primary, secondary, acute or convalescent. A sample was defined as dengue if it was positive for at least one of dengue IgM, IgG or NS1 antigen biomarker and non-dengue if negative for all three biomarkers. A primary dengue sample was one in which the IgM/IgG optical density (OD) ratio is ≥1.2. If the ratio was <1.2 it was designated as secondary [12, 20, 21]. Sample collection time was expressed in days(s) post onset of symptoms (DPO) and was interpreted with day 1 being the first 24 h. An acute sample was one with a DPO ≤ 5 days while a convalescent sample was defined as having a DPO > 5 days. The combination strategies for the SDB DD ICT are as described before [22]. An IgM/NS1 combination result meant dengue if either IgM or NS1 was positive and non-dengue if both were negative irrespective of the IgG result. The IgM/NS1/IgG combination result was interpreted as dengue if at least one of SD IgM, IgG or NS1 was positive and non-dengue if all three were negative.

### Statistical analysis

Microsoft Excel (Microsoft Inc., Redmond, Washington, USA) was used for data entry and data analysis was done using Epi info 7 (Centers for Disease Control and Prevention, Atlanta, USA). Chi-square test and the Fisher’s exact test (two sided) were used to compare categorical variables. A probability value (*p*) of < 0.05 was considered statistically significant. The sensitivity, specificity, positive predictive value (PPV), negative predictive value (NPV) and kappa coefficient were calculated as previously described [23, 24].

## Results

### Patients’ samples

The 339 consecutively archived samples selected for the evaluation were obtained from 159 (47 %) female and 180 (53 %) males. The median age was 13 years (range, 10 days to 95 years) and the median time between the onset of illness and collection of specimens was 4 days (range, 1 to 22 days). The samples were characterized using the reference ELISAs for dengue IgM, IgG and NS1 antigen and classified initially as 309 (91 %) dengue and 30 (9 %) non-dengue. The 309 dengue samples were further defined by dengue phase as 189 (61 %) acute, 120 (39 %) convalescent and by immune status as 80 (26 %) primary and 229 (74 %) secondary.

### Overall diagnostic performance

The diagnostic performance of the individual assay parameters in comparison to the reference standard ELISAs is shown in Table 1. The overall diagnostic sensitivities of the SDB DD rapid test for IgM, IgG, and NS1 were 49.3 % (95 % CI: 41.3-57.4), 39.1 % (95 % CI 33.3-45.2) and 90 % (95 % CI: 82.1-94.7), respectively. The IgM and IgG detection rates were significantly lower than that of the NS1 (*p* < 0.001). However, the combination of the IgM detection with NS1 detection or both NS1 and IgG resulted in a significant (*p* < 0.001) increase in sensitivity to 97.5 % (95 % CI: 92.9-99.2) and 98.9 % (95 % CI: 96.0-99.7), respectively. These higher sensitivities were achieved with increase specificities of 100 %. The kappa coefficients for the NS1, IgM/NS1 and IgM/NS1/IgG parameters were 0.91 (95 % CI: 0.81-1.0), 0.97 (95 % CI: 0.86-1.0), and 0.96 (95 % CI: 0.83-1.0), respectively.

**Table 1:** Overall diagnostic performance of the SDB DD ICT against reference ELISAs

Dengue Parameter	No. of dengue ELISA pos/neg	No. of SDB DD ICT pos/neg	Sensitivity % (95 % CI)	Specificity % (95 % CI)	PPV % (95 % CI)	NPV % (95 % CI)
IgM	145/194	71/187	49.3 (41.3-57.4)	95.9 (92.1-97.9)	89.9 (81.3-94.8)	71.9 (66.2-77.0)
NS1	90/249	81/247	90.0 (82.1-94.7)	99.2 (97.1-99.8)	97.6 (91.6-99.3)	96.5 (93.5-98.1)
IgG	256/83	100/83	39.1 (33.3-45.2)	100 (95.6-100)	100 (96.3-100)	34.7 (29.0-41.0)
IgM/NS1	121/157	117/157	97.5 (92.9-99.2)	100 (97.6-100)	100 (96.8-100)	98.1 (94.6-99.4)
IgM/ NS1/ IgG	181/29	179/29	98.9 (96.0-99.7)	100 (88.3-100)	100 (97.9-100)	93.6 (79.3-98.2)

95 % CI is shown in parenthesis. pos = positive; neg = negative; SDB DD = SD BIOLINE Dengue DUO®; ICT = Immunochromatographic test

The IgG test had the lowest sensitivity of 39.1 % (95 % CI: 33.3-45.2) but the highest individual specificity of 100 % (95 % CI: 95.6-100). It also demonstrated a very high individual positive predictive value (PPV) of 100 % (95 % CI: 95.6-100) but a very low negative predictive value (NPV) of 34.7 % (95 % CI: 29.0-41.0). The PPV and NPV for the NS1 component, the combined IgM/NS1 and IgM/NS1/IgG were all high.

### Sensitivity of SDB DD tests according to primary and secondary dengue immune status

**Fig. 1:**
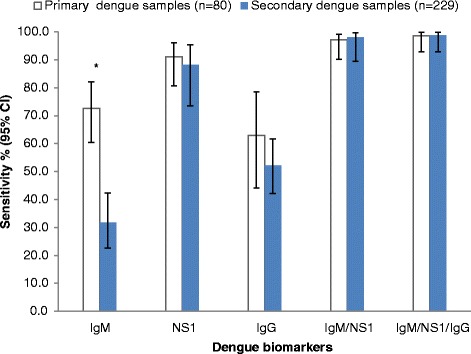
Sensitivity of SDB DD ICT according to dengue immune status. Data represent sensitivity (%) and error bars represent 95 % CI. *indicates significance at p < 0.001, when comparing primary and secondary dengue samples

Figure 1 shows that the IgM detection was significantly higher in samples with primary dengue (72.6 %; 95 % CI: 60.4-82.1 %) than secondary dengue (31.7 %; 95 % CI: 22.7-42.4) infection (*p* < 0.001). Although the sensitivities of the NS1 and IgG markers were lower in secondary dengue samples (88.2 % and 52.1 %, respectively), than in primary (91.1 % and 63.0 %, respectively), the differences were not significant. There was a slight non-significant increase in the sensitivities of the IgM/NS1 and IgM/NS1/IgG combined dengue biomarkers in secondary dengue samples (98 %; 95 % CI: 89.5-99.7 and 98.6 %; 95 % CI: 92.9-99.8, respectively), over primary dengue samples (97.1 %; 95 % CI: 90.2-99.2 and 98.6 %; 95 % CI: 92.3-99.0, respectively).

### Sensitivity of SDB DD tests according to acute and convalescent phases

**Fig. 2:**
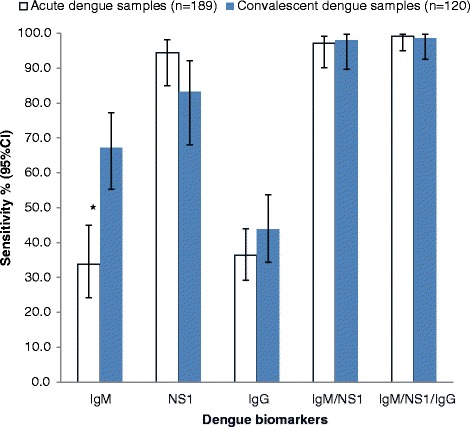
Sensitivity of SDB DD ICT according to sample collection phase. Data represent sensitivity (%) and error bars represent 95 % CI. *indicates significance at the p < 0.001 level when comparing acute and convalescent dengue samples

The IgM was significantly more sensitive (*p* < 0.001) in convalescent serum samples (67.2 %; 95 % CI: 55.3-77.2) as compared to acute samples (33.8 %; 95 % CI: 24.2-44.9) (Fig. 2). In contrast, the sensitivity of the IgG, NS1, and the combined strategies of IgM/NS1 and IgM/NS1/IgG were not significantly affected by acute or convalescent dengue phases.

### Sensitivity of SDB DD NS1 test according to IgM/IgG antibody profiles

In this study, the presence of IgM and/or IgG did not significantly impact the sensitivities of the SDB DD NS1 tests although the sensitivities generally trend higher in the absence of IgM/IgG (Table 2). The sensitivities of the SDB DD dengue biomarkers were not affected by age or sex.

**Table 2:** Sensitivity of the SDB DD NS1 parameter according to IgM/IgG antibody profile

Dengue antibody profile	Sensitivity % (95 % CI)	N	P value
IgM^−^/IgG^−^	94.4(74.2- 99.0)	18	0.899^a^
IgM^+^/IgG^−^	91.2(77.0-97.0)	34	
IgM^−^/IgG^+^	100(75.8-100)	12	0.265^b^
IgM^+^/IgG^+^	80.8(62.1-91.5)	26	

95 % CI is shown in parenthesis; − = negative; + = positive; a = comparison between IgM^−^/IgG^−^ and IgM^+^/IgG^−^ ; b = comparison between IgM^−^/IgG^+^ and IgM^+^/IgG^+^

## Discussion

The NS1 test component of the SDB DD ICT kit had the highest individual sensitivity of 90 % when compared to the reference ELISA for the diagnosis of dengue in Jamaica. Slightly lower but similarly high sensitivities (81.6 % and 87.5 %) were also reported by Gan *et al.* in Singapore [25] and by Sanchez-Vargas *et al.* in Mexico [26], respectively. In both of those studies the authors used a different NS1 ELISA (Platelia™) as comparator.

In contrast, others have reported markedly lower SDB DD NS1 sensitivity findings in the range of 44-51 % [22, 27, 28]. The lower sensitivities were attributed to the possible presence of high IgG antibody titres in those samples. It was hypothesized that dengue viral antigens, inclusive of NS1, may form immune complexes with high levels of dengue IgG antibodies and thus become undetectable [29, 30]. High titres of IgG are more likely to be found in secondary dengue infections. Andries *et al.* [28], for example, found significant reduction of NS1 sensitivity in secondary infection (43.4 %) when compared to primary infection (89.5 %) (*p* < 0.001).

While the sensitivity of the SDB DD NS1 in this study was generally lower in the presence of measurable IgG antibodies this was not statistically significant. Similarly, although we found a lower NS1 sensitivity in secondary infections than in primary infections, this again was not significant. Our results, however, are not unique and similar findings were reported by Gan *et al.* and Sanchez-Vargas *et al.* [25, 26].

It appears therefore that although IgG antibody titres may have some impact on the NS1 sensitivity levels, this alone may not explain the low sensitivity levels obtained in some studies [22, 27, 28]. Perhaps, other factors including the type of reference method used in the comparisons were contributing to the observed trends. Those studies with low NS1 sensitivities all used non-NS1 comparators such as virus isolation and genome detection by polymerase chain reaction (PCR) as the reference standards [22, 27, 28]. Hang *et al.* and Tricou *et al.* in their evaluations of the relationship between NS1 sensitivity to viraemia using several NS1 assays have shown that NS1 levels correlate with viraemia [30, 31]. They have also found that NS1 negative patients had significantly lower mean viraemia than NS1 positive patients. Although the correlation between NS1 detection and viraemia is not precise, we are of the opinion that studies which use virus isolation or genome detection methods may be detecting lower levels of viraemia in those samples which, in effect, are less likely to be NS1 positive.

The fact that the SD NS1 sensitivity, in our study, was high and was not significantly affected by factors such as primary/secondary infection status, acute/convalescent and the presence/absence of IgM/IgG antibodies is definitely advantageous. Accordingly, this test is ideal for use in dengue endemic as well as non-endemic countries. It would also be extremely helpful in making dengue diagnosis in areas where patients present in either the acute (≤5 days) and early convalescent (>5 days) phases of dengue illness.

The overall sensitivity of the SDB DD IgM component alone was low and is consistent with a previously published report of a sensitivity of 47 % and 49.7 % in Puerto Rico and Malaysia, respectively [17]. Clinicians should, therefore, not use a negative IgM result alone to rule out dengue infection in these settings. The SDB DD dengue IgM test parameter was significantly lower in acute and secondary dengue samples. These are expected findings which demonstrate the dynamics of the host IgM immune response [29, 32]. IgM is therefore considered more a marker of recent infection rather than of acute infection [29] and so this limits its usefulness when used alone as a single biomarker in the diagnosis of dengue infection.

The limitations of the SDB DD IgM biomarker alone, however, disappeared when it was combined with other dengue biomarkers. Herein lies the advantage of the SDB DD kit in that it has the capacity to measure both dengue antigen and dengue antibodies in one test. This allows for different testing strategies. We explored different combination approaches with the IgM/IgG and NS1 biomarkers and found that combining any two or more of the individual components resulted in even greater levels of diagnostic sensitivities. Our data provides evidence, in keeping with others who have recently reported, that using a combination strategy enhances the overall diagnostic performance of rapid dengue diagnostic kits [19, 25–28, 33]. For example, Wang *et al.* [19] reported improvement in sensitivity from 53.5 % (IgM alone) to 88.7 % (NS1/IgM) while Sanchez-Vargas *et al.* [26] found enhanced sensitivity from 60.51 % (IgM alone) to 90.65 % (NS1/IgM/IgG). It should be noted that in both of the above mentioned studies the improvement in sensitivities came with slight decrease in specificities unlike our study in which there were slight increase in specificities to 100 %. This pattern of improvement in test performance by combination of multiple dengue biomarkers is not only observed for SDB DD ICT kits but also for other rapid dengue diagnostic kits [25, 33]. Additionally, in the current study, the sensitivity of the combination of the NS1/IgM/IgG biomarkers was not significantly affected by dengue immune and phase status which is in agreement with others [25, 28].

There are some limitations in the current study. Firstly, the study samples were not specifically tested for other flaviviruses nor for other diseases such as malaria and leptospirosis, and therefore it is not known what level of cross reactivities were present. It would have been interesting to demonstrate the effect, if any, of West Nile virus (WNV) infection on the specificities of the SDB DD ICT kit since a previous study by Brown *et al.* (unpublished data) among dengue suspected cases in Jamaica showed that 5.9 % (26/435) had cross reactive antibodies to WNV.

Secondly, there could be misclassification bias due to: (1) use of single serum samples which would not be able to demonstrate dengue seroconversion as may occur with paired samples and (2) use of serological tests alone instead of more sensitive methods of detection e.g. PCR. Further studies are recommended to investigate the effect of disease severity, other viral serotypes and comparative molecular testing methods on the performance of the SDB DD ICT kit in Jamaica.

## Conclusions

In summary, this evaluation showed that the NSI component of the SDB DD ICT kit is very sensitive and specific for the diagnosis of dengue infections irrespective of samples being acute/convalescent or primary/secondary. Combining the IgM parameter with detection of either NS1 alone or both NS1 and IgG significantly improved its sensitivity without decreasing the specificity. The SDB DD ICT kit is therefore a very useful tool for the diagnosis of dengue.

## References

[1] Ranjit S, Kissoon N, Shamarao S (2009). Dengue viral infections and shock syndromes: an overview. J Ped Infect Dis.

[2] Ross TM (2010). Dengue virus. Clin Lab Med.

[3] Normile D (2013). Surprising new dengue virus throws a spanner in disease control efforts. Science.

[4] McBride WJ, Bielefeldt-Ohmann H (2000). Dengue viral infections; pathogenesis and epidemiology. Microbes Infect.

[5] Senanayake S (2006). Dengue fever and dengue haemorrhagic fever—a diagnostic challenge. Aust Fam Physician.

[6] Halstead SB (2007). Dengue. Lancet.

[7] Brown MG, Vickers IE, Salas RA, Smikle MF (2009). Seroprevalence of dengue virus antibodies in healthy Jamaicans. Hum Antibodies.

[8] Dick OB, San Martin JL, Montoya RH, del Diego J, Zambrano B, Dayan GH (2012). The history of dengue outbreaks in the Americas. Am J Trop Med Hyg.

[9] Pan American Health Organization Wkly Epidemiol Rep, 2012. Number of reported cases of dengue and severe dengue (SD) in the Americas, by country- 15 December 2012 (EW48). Available at: http://www.paho.org/hq/index.php?option=com_docman&task=doc_view&gid=19582&Itemid=2518.

[10] Moncayo AC, Baumblatt J, Thomas D, Harvey KA, Atrubin D, Stanek D (2015). Dengue among American Missionaries Returning from Jamaica, 2012. Am J Trop Med Hyg.

[11] Kalayanarooj S (2011). Clinical Manifestations and Management of Dengue/DHF/DSS. Trop Med Health.

[12] WHO (2009). Dengue: guidelines for the diagnosis, treatment, prevention and control- new edition.

[13] Gregory CJ, Lorenzi OD, Colón L, Garcìa AS, Santiago LM, Rivera RC (2011). Utility of the tourniquet test and the white blood cell count to differentiate dengue among acute febrile illnesses in the emergency room. PLoS Negl Trop Dis.

[14] Guzmàn MG, Kouri G (2004). Dengue diagnosis, advances and challenges. Int J Infect Dis.

[15] Groen J, Koraka P, Velzing J, Copra C, Osterhaus AD (2000). Evaluation of six immunoassays for detection of dengue virus-specific immunoglobulin M and G antibodies. Clin Diagn Lab Immunol.

[16] Wang SM, Sekaran SD (2010). Evaluation of a commercial SD dengue virus NS1 antigen capture enzyme linked immunosorbent assay kit for early diagnosis of dengue viru infection. J Clin Microbiol.

[17] Hunsperger EA, Yoksan S, Buchy P, Nguyen VC, Sekaran SD, Enria DA (2009). Evaluation of commercially available anti-dengue virus immunogobulin M tests. Emerg Infect Dis.

[18] Nga TT, Thai KT, Phuong HL, Giao PT, le Hung Q, Binh TQ (2007). Evaluation of two rapid immunochromatographic assays for diagnosis of dengue among Vietnamese febrile patients. Clin Vaccine Immunol.

[19] Wang SM, Sekaran SD (2010). Early diagnosis of dengue infection using a commercial Dengue Duo rapid test kit for the detection of NS1, IgM, and IgG. Am J Trop Med Hyg.

[20] Shu PY, Chen LK, Chang SF, Yueh YY, Chow L, Chien LJ (2003). Comparison of capture Immunglobulin M (IgM) and IgG enzyme-linked immunosorbent assay (ELISA) and nonstructural protein NS1 serotype-specific IgGELISA for differentiation of primary and secondary dengue virus infections. Clin Diagn Lab Immunol.

[21] Falconar AK, de Plata E, Romero-Vivas CM (2006). Altered enzyme-linked immunosorbent assay immunoglobulin M (IgM) /IgG optical density ratios can correctly classify all primary or secondary dengue virus infections 1 day after the onset of symptoms, when all of the viruses can be isolated. Clin Vaccine Immunol.

[22] Osorio L, Ramirez M, Bonelo A, Villar LA, Parra B (2010). Comparison of the diagnostic accuracy of commercial NS1-based diagnostic tests for early dengue infection. Virol J.

[23] Lalkhen AG, McCluskey A (2008). Clinical tests: sensitivity and specificity. Critical Care & Pain J.

[24] Landis JR, Koch GG (1977). The measurement of observer agreement for categorical data. Biometrics.

[25] Gan VC, Tan LK, Lye DC, Pok KY, Mok SQ, Chua RC (2014). Diagnosing dengue at the point-of-care: utility of a rapid diagnostic kit in Singapore. PLoS One.

[26] Sánchez-Vargas LA, Sánchez-Marce EE, Vivanco-Cid H (2014). Evaluation of the SD BIOLINE Dengue Duo rapid test in the course of acute and convalescent dengue infection in a Mexican endemic region. Diagn Microbiol Infect Dis.

[27] Blacksell SD, Jarman RG, Bailey MS, Tanganuchitcharnchai A, Jenjaroen K, Gibbons RV (2011). Evaluation of six commercial point-of-care tests for diagnosis of acute dengue infections: the need for combining NS1 antigen and IgM/IgG antibody detection to achieve acceptable levels of accuracy. Clin Vaccine Immunol.

[28] Andries AC, Duong V, Ngan C, Ong S, Huy R, Sroin KK (2012). Field evaluation and impact on clinical management of a rapid diagnostic kit that detects dengue NS1, IgM, and IgG. PLoS Negl Trop Dis.

[29] Blacksell SD (2012). Commercial Dengue rapid diagnostic tests for point-of-care application: recent evaluation and future needs?. J Biomed Biotechnol.

[30] Hang VT, Nguyet NM, Trung DT, Tricou V, Yoksan S, Dung NM (2009). Diagnostic accuracy of NS1 ELISA and lateral flow rapid tests for dengue sensitivity, specificity and relationship to viraemia and antibody responses. PLoS Negl Trop Dis.

[31] Tricou V, Vu HT, Quynh NV, Nguyen CV, Tran HT, Farrar J (2010). Comparison of two dengue NS1 rapid tests for sensitivity, specificity and relationship to viraemia and antibody responses. BMC Infect Dis.

[32] Shu PY, Huang JH (2004). Current advances in dengue diagnosis. Clin Diagn Lab Immunol.

[33] Fry SR, Meyer M, Semple MG, Simmons CP, Sekaran SD, Huang JX (2011). The diagnostic sensitivity of dengue rapid test assays is significantly enhanced by using a combined antigen and antibody testing approach. PLoS Negl Trop Dis.

